# Evaluation Strategies for Understanding Experiences With Virtual Care in Canada: Mixed Methods Study

**DOI:** 10.2196/45287

**Published:** 2023-08-30

**Authors:** Shelley Vanderhout, Ellen B Goldbloom, Amy Li, Dennis Newhook, Meghan Garcia, Catherine Dulude

**Affiliations:** 1 Children’s Hospital of Eastern Ontario Research Institute Ottawa, ON Canada; 2 Faculty of Medicine University of Ottawa Ottawa, ON Canada; 3 Children’s Hospital of Eastern Ontario Ottawa, ON Canada

**Keywords:** environmental scan, experience, interviews, pediatrics, telemedicine, virtual care

## Abstract

**Background:**

Virtual care was rapidly integrated into pediatric health services during the COVID-19 pandemic. While virtual care offers many benefits, it is necessary to better understand the experiences of those who receive, deliver, and coordinate virtual care in order to support sustainable, high-quality, and patient-centered health care. To date, methods implemented to evaluate users’ experiences of virtual care have been highly variable, making comparison and data synthesis difficult.

**Objective:**

This study aims to describe evaluation strategies currently used to understand personal experiences with pediatric virtual care in Canada.

**Methods:**

In this mixed methods environmental scan, we first distributed a web-based questionnaire to clinical, research, and operational leaders delivering and evaluating pediatric virtual care in Canada. The questionnaire collected information about how experiences with virtual care have been or are currently being evaluated and whether these evaluations included the perspectives of children or youth, families, providers, or support staff. Second, respondents were asked to share the questions they used in their evaluations, and a content analysis was performed to identify common question categories. Third, we conducted semistructured interviews to further explore our respondents’ evaluation experiences across 4 domains—evaluation approaches, distribution methods, response rates, and lessons learned—and interest in a core set of questions for future evaluations.

**Results:**

There were 72 respondents to the web-based questionnaire; among those who had conducted an evaluation, we identified 15 unique evaluations, and 14 of those provided a copy of the tools used to evaluate virtual care. These evaluations measured the virtual care experiences of parents or caregivers (n=15, 100%), children or youth (n=11, 73%), health care providers (n=11, 73%), and support staff (n=4, 27%). The most common data collection method used was electronic questionnaires distributed by email. Two respondents used validated tools; the remainder modified existing tools or developed new tools. Content analysis of the 14 submitted questionnaires revealed that the most common questions were about overall participant satisfaction, the comparison of virtual care to in-person care, and whether participants would choose virtual care options in the future. Interview findings indicate respondents frequently relied on methods used by peers and that a standardized, core set of questions to evaluate experiences with virtual care would be helpful to improve evaluation practices and support pediatric health care delivery.

**Conclusions:**

At our institution and elsewhere in Canada, experiences with pediatric virtual care have been evaluated using a variety of methods. A more consistent evaluation approach using standardized tools may enable more regular comparisons of experiences with virtual care and the synthesis of findings across health care settings. In turn, this may better inform our approach to virtual care, improve its integration into health systems, and facilitate sustainable, high-quality, patient-centered care.

## Introduction

Virtual care is defined as any interaction between patients and members of their circle of care using technologies and applications that support synchronous (eg, videoconferencing and telephone) or asynchronous (eg, email and messaging through the patient portal) health care delivered at a distance [1-5]. Virtual care (including telehealth and telemedicine [3]) rapidly evolved with the onset of the COVID-19 pandemic. In many health care settings, virtual care temporarily replaces a significant proportion of in-person encounters. There are a number of reported benefits of virtual care, including convenience, cost savings, improved accessibility, and higher patient satisfaction [6-9]. Specific to pediatrics, many families report that virtual care is also less disruptive to their daily routines [10]. Despite some drawbacks, such as the inability to physically examine patients and the potential for technical difficulties, virtual care is likely to remain an option beyond the COVID-19 pandemic. As such, various aspects of virtual care services and programs need to be evaluated to measure their safety and effectiveness toward enhancing pediatric health outcomes [2,11]. Various frameworks are available to guide the evaluation of virtual care, and most recommend including the experiences of patients, families, providers, and support staff as a core measurement domain [12-14]. As recognized by the Quintuple Aim [15], it is essential to understand the experiences not only of patients and caregivers who receive virtual care but also of health care providers and support staff who deliver and coordinate it for optimal health system performance.

Despite recommendations to include the perspectives of all users, the majority of telehealth assessment tools presently available in the literature capture only patient and family feedback, and few have been developed to include provider perspectives. There is a lack of pediatric-focused tools and measurement of patient or family demographic information. Enhanced demographic data could add valuable insight to our understanding of a population’s telehealth use. For instance, socioeconomic status or place of residence may relate to access to technology and, therefore, virtual care experience [5,13,16,17].

Given the pandemic-expedited increase in virtual care and the need to better understand how best to evaluate virtual care to ensure data-informed health system changes, we conducted an environmental scan of how experiences with virtual care are evaluated within Canadian pediatric institutions. The objective of this scan was to use quantitative and qualitative methods to describe the various evaluation strategies (including their methods, tools, and implementation effectiveness) for measuring the experiences of children and youth, parents and caregivers, health care providers, and support staff with synchronous pediatric virtual care in Canada. In addition, we sought to describe a core set of evaluation questions that may support the comparison and synthesis of data within and across pediatric virtual care programs and contexts over time.

## Methods

### Overview

We used a mixed methods approach to our environmental scan, using a web-based questionnaire to gather information about virtual care evaluations, content analysis of collected evaluation tools, and semistructured interviews. This study is reported in accordance with the CHERRIES (Checklist for Reporting Results of Internet E-Surveys; Multimedia Appendix 1) [18] and the GRAMMS (Good Reporting of Mixed Methods Study) framework [19]. Study team members (EG, MG, and CD) drafted the questionnaire based on subject matter expertise, existing literature, peer consultation, and the operational needs of the study team’s institution. We revised the tool based on advice from a research methodologist experienced in survey design and pilot feedback from 3 virtual care leaders representing pediatric research, clinical care, and operations. We distributed the finalized questionnaire (see Multimedia Appendix 2) through REDCap [20], which was open and did not require a password to access. The final version contained 11 items, displayed on 1 continuous page, to collect information about respondents’ current (or previous) virtual care evaluation activities, including their evaluation participants, methods, and response rates. Respondents were able to change their answers before submitting them. We also asked for participant consent to be contacted to provide copies of their evaluation tools and participate in a follow-up interview. There were no incentives offered to participate.

The study population included clinical, research, and operational staff who were delivering and evaluating synchronous virtual care in a Canadian pediatric context. We recruited respondents using 2 methods. Locally, we invited clinical and operational leaders within our institution through email (see Multimedia Appendix 3) to complete the questionnaire or to forward the email to a colleague better suited to complete it. This email explained that participation was voluntary, provided a link to access the electronic questionnaire through REDCap, and summarized the contents of the information letter that was presented at the beginning of the electronic questionnaire for participants to read before answering the questions. We sent 3 reminder emails following the initial invitation. To reach a national audience, we asked virtual care leaders within our organization to forward the email to people in their networks across Canada who may have been eligible to participate and promoted the study on social media through Children’s Healthcare Canada, a national association enabling improvements in children’s health care [21]. The questionnaire was active for 6 weeks, from December 2021 to January 2022.

The electronic questionnaire was built in REDCap, and all data were stored on a password-protected server. We analyzed response data descriptively using frequencies. We also reviewed responses to identify and remove duplicates (ie, multiple respondents reporting on the same evaluation), and in cases where consent to contact had been provided, we verified potential duplicates with respondents. The questions from the evaluation tools that we received were entered into a spreadsheet for content analysis [22]. Questions that were not related to virtual care experience (eg, “Approximately how often are you seen in the neurology clinic?” or “Did you receive any other services outside of [our institution]?”) were excluded from the analysis. With regular feedback from the study team, SV iteratively developed question categories that reflected the content of the questionnaires and unique aspects of virtual care and tabulated the number of questions in each category. CD and DN independently reviewed the question categories and verified that all questions were appropriately categorized.

To integrate qualitative and quantitative analyses, we invited respondents who had conducted evaluations of virtual care and provided consent to be contacted for follow-up to participate in an individual 30-minute semistructured interview (see Multimedia Appendix 4). Two members of the research team, SV and CD, conducted the interviews virtually using Microsoft Teams. We used purposive sampling to ensure interview participants represented varied clinical areas and evaluation approaches. We developed a general interview guide, which we tailored to each respondent based on their questionnaire responses, to gain further insight into participants’ experience of 4 domains: evaluation approaches, distribution methods, response rates, and lessons learned. We also sought feedback on participants’ interest in a core set of questions, which could be constructed according to the categories we developed from analyzing evaluation tools and used to evaluate experience with virtual care in the future. The interviews were recorded and transcribed for content analysis [22]. The analysis, led by SV, was predominantly deductive, whereby the transcripts were coded using the same a priori domains of interest. Exemplary quotes were selected from the interviews to enrich our understanding of each domain.

### Ethics Approval

This study was approved by the research ethics board at the Children’s Hospital of Eastern Ontario (CHEO; IRB #1, IRB00002747, protocol #21/112X), and all respondents consented to participate.

## Results

### Questionnaire

**Figure 1:**
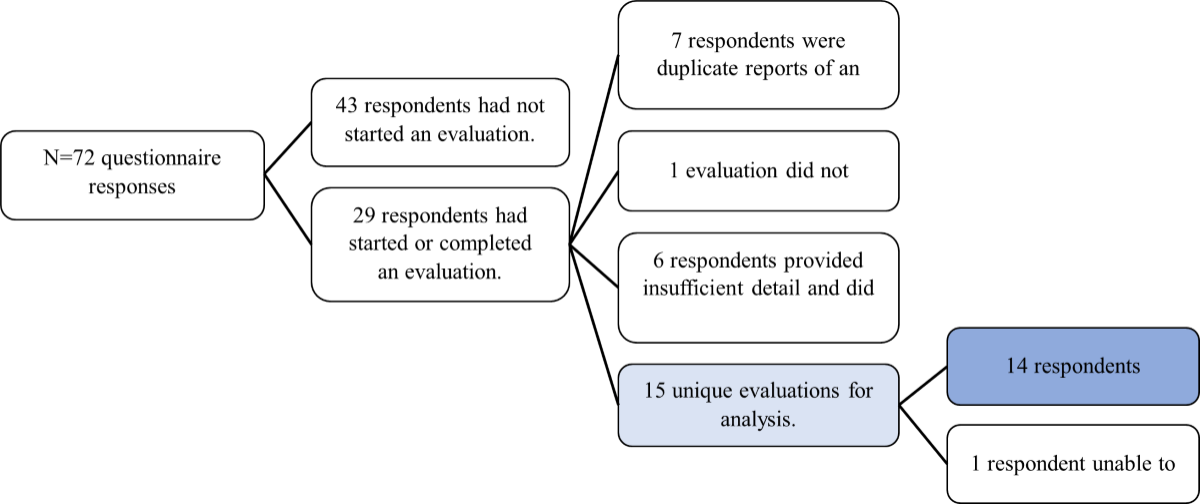
Virtual care experience evaluations conducted by questionnaire respondents.

There were 72 respondents to our web-based questionnaire. The majority of respondents (n=65, 90%) were in Ontario; others were from across Canada, including Alberta, British Columbia, Nova Scotia, and Saskatchewan. Most respondents (n=68, 94%) were affiliated with hospitals, apart from 2 (3%) who represented provincial health authorities and another 2 (3%) who were affiliated with academic institutions. Most held clinician roles (n=50, 69%), while others were program leads, managers, and researchers; some respondents held multiple roles. Respondents represented virtual care programs in ambulatory care or outpatient medical clinics most often (n=30, 42%), followed by emergency department or urgent care (n=13, 18%), inpatient medicine (n=13, 18%), and outpatient mental health (n=12, 17%). Respondent characteristics are shown in Table 1. Overall, 29 (40%) respondents had conducted or were currently conducting an evaluation of experiences with virtual care at the time of data collection (Figure 1). Of these, we identified 15 unique evaluations (see Tables 2 and 3 and Textbox 1) that measured the experiences of parents or caregivers (n=15, 100%), children and youth (n=11, 73%), health care providers (n=11, 73%), and support staff (n=4, 27%). The most common evaluation tools used were questionnaires. Two (13%) of the 15 evaluations used validated tools (the Telehealth Usability Questionnaire [TUQ] [23] and the Measure of Processes of Care-20 [24]); the others used modified tools (n=2, 13% modified the TUQ; n=2, 13% modified nonvalidated tools) or, most commonly, original tools (n=9, 60%). Few evaluations used focus groups or interviews. In most cases, evaluations were distributed through email, followed by patient portals, phone, or word of mouth. Response rates ranged from 20% to 100% and were reported for 4 (36%) of the 11 evaluations conducted among children and youth, 5 (33%) of the 15 evaluations conducted with parents and caregivers, 3 (27%) of the 11 evaluations conducted with providers, and 1 (25%) of the 4 evaluations conducted with support staff. Most of the time, these data were missing or respondents did not know.

**Table 1:** Survey respondent characteristics (N=72).

Characteristic		Respondents, n (%)
**Province**		
	Alberta	1 (1)
	British Columbia	3 (4)
	Ontario	65 (90)
	Nova Scotia	1 (1)
	Saskatchewan	1 (1)
	Missing	2 (3)
**Role^a^**		
	Director	6 (8)
	Manager	10 (14)
	Program lead	22 (31)
	Clinician	50 (69)
	Administration	8 (11)
	Research	10 (14)
	Other	4 (6)
**Areas where respondents work, and virtual care was provided^a^**		
	Autism	4 (6)
	Critical care	4 (6)
	Emergency department or urgent care	13 (18)
	Genetics	4 (6)
	Inpatient development or rehabilitation	2 (3)
	Inpatient medicine	13 (18)
	Inpatient mental health	6 (8)
	Outpatient development or rehabilitation	9 (13)
	Outpatient medical clinic or ambulatory care	30 (42)
	Outpatient mental health	12 (17)
	Perioperative services	7 (10)
	Other	9 (13)
**A virtual care experience evaluation is underway or complete**		
	Yes	29 (40)
	No	43 (60)

^a^More than one selection possible.

**Table 2:** Evaluation characteristics (n=15).

Characteristic			Participants, n (%)
**Province**			
	Alberta	1 (7)	
	British Columbia	1 (7)	
	Ontario	12 (75)	
	Nova Scotia	1 (7)	
**Evaluation setting**			
	Autism	1 (7)	
	Critical care	1 (7)	
	Emergency department or urgent care	3 (15)	
	Outpatient development or rehabilitation	2 (13)	
	Outpatient mental health	2 (13)	
	Palliative care	1 (7)	
	Perioperative services	1 (7)	
	Diabetes	2 (13)	
	Complex care	1 (7)	
	Respirology	1 (7)	
	Neurology	1 (7)	
**Evaluation population**			
	Children or youth	11 (73)	
	Parents or caregivers	15 (100)	
	Health care providers	11 (73)	
	Support staff	4 (27)	

**Table 3:** Characteristics of virtual care evaluations.

				Children or youth, n (%)		Parents or caregivers, n (%)		Health care providers, n (%)			Support staff, n (%)
**Evaluation method^a^**											
	**Survey**		11 (100)		15 (100)		9 (82)			2 (50)	
		Validated	1 (9)		2 (13)		1 (11)			0 (0)	
		Nonvalidated	10 (91)		13 (87)		8 (89)			2 (100)	
	Focus group		1 (9)		0 (0)		0 (0)			0 (0)	
	Interview		2 (18)		5 (33)		5 (45)			2 (50)	
**Type of tool used^a^**											
	Existing tool		1 (9)		2 (13)				1 (9)	0 (0)	
	Added to or modified an existing tool		3 (27)		5 (33)				2 (18)	1 (25)	
	Developed own tool		7 (64)		8 (53)				8 (73)	3 (75)	
**Distribution method^a^**											
	Email		11 (100)		13 (87)		11 (100)			4 (100)	
	Patient portal		2 (18)		2 (13)		1 (9)			0 (0)	
	Phone call		2 (18)		3 (20)		1 (9)			0 (0)	
	Word of mouth		0		3 (20)		1 (9)			1 (25)	
**Response rate**											
	0%-20%		0 (0)		0 (0)		0 (0)			0 (0)	
	21%-40%		2 (18)		2 (13)		0 (0)			0 (0)	
	41%-60%		0 (0)		0 (0)		1 (9)			0 (0)	
	61%-80%		0 (0)		2 (13)		0 (0)			0 (0)	
	81%-100%		2 (18)		1 (7)		2 (18)			1 (25)	
	I don’t know		1 (9)		3 (20)		3 (27)			0 (0)	
	Missing		6 (55)		7 (47)		5 (45)			3 (75)	

^a^More than one selection possible.

**Considerations for evaluating experiences with virtual care.**

**Considerations for evaluation methods:**

- 1-time surveys may yield better response rates than those that follow participants over time, which introduce the risk of attrition.

**Considerations for tool selection:**

- Literature searches, environmental scans, and consultation with colleagues can help identify existing tools, which can be combined with program-specific questions to suit unique clinical contexts.
- Some tools have versions available for both providers and patients or families, which can aid data analysis.

**Considerations for determining a distribution method:**

- Sending evaluations through email to patients and families within a day of the virtual encounter may help gather visit-specific information.
- Paper evaluations may not be effective, as patients and their families are likely not attending care in person.
- Recruitment through email may enable linkage to health records with consent, which can add demographic information to evaluation responses.
- For providers, in-person recruitment methods may be more effective than email due to the possibility of emails being missed.

**Considerations for evaluating response representativeness:**

- If possible, comparing the demographics of program-specific evaluation participants to the broader patient population can help determine response representativeness.

### Content Analysis of Evaluation Tools

**Figure 2:**
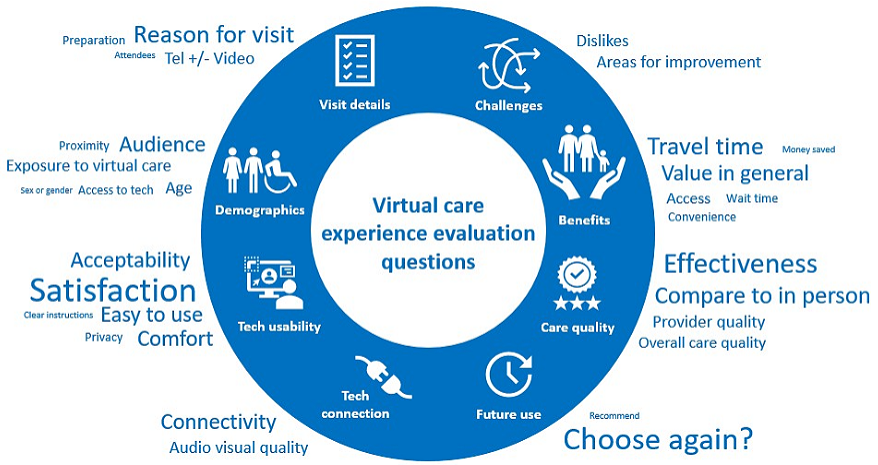
Emergent question categories and category domains identified through content analysis of submitted virtual care experience evaluation tools. Categories identified in at least 3 of the 15 submitted tools are presented outside the circle, with increased frequency reflected by larger category text size. Domains, representing a group of related categories, are identified with an icon, and presented inside the circle.

Of the 15 unique evaluations, 14 participants submitted their evaluation tools (1 could not be shared due to copyright). Content analysis revealed the total number of questions asked ranged from 3 to 26, with an average of 15.4 (SD 7.6) questions. Of the 14 evaluations, 10 (71%) included at least one demographic question. Likert scales were the most common response scale used; 10 (71%) tools used Likert scales ranging from 4- to 7-points in at least one question, 8 (57%) tools included at least one closed-ended question, and 8 (57%) tools included at least one open-ended question. Excluding demographics and visit details, content analysis revealed 24 question categories exploring experiences with virtual care (Figure 2 and Table 4). Examples of questions in each category are found in Multimedia Appendix 5. The most common categories were overall participant satisfaction (11/14, 79% of tools asked a question in this category), comparison of virtual care to in-person care (11/14, 79%), and likelihood to attend a future virtual care visit (10/14, 71%). Few (1/14, 7%) tools asked about ethnicity or race, education level, or employment status. We compared the 24 question categories to the items included in 2 commonly used and validated questionnaires about virtual care experience, the TUQ [23] and Telehealth Satisfaction Questionnaire (TSQ; Table 4) [25]. A total of 9 of our categories matched questions from both questionnaires, 11 of our categories did not match any questions from these questionnaires, and 4 matched a question from 1 questionnaire.

**Table 4:** Comparison of emergent question categories (excluding demographics and visit details) to the Telehealth Satisfaction Questionnaire (TSQ) [25] and Telehealth Usability Questionnaire (TUQ) [23].

Emergent question categories	Emergent questions, n (%)	TSQ	TUQ
Travel time	8 (57)	Telemedicine saves me time traveling to hospital or a specialist clinic.	Telehealth saves me time traveling to a hospital or specialist clinic.
Money saved	3 (21)	—^a^	—
Convenience	4 (29)	—	—
Wait time (day of appointment)	4 (29)	—	—
Access to care	5 (36)	I obtain better access to health care services by use of telemedicine, and I meet with my health care provider more frequently through telemedicine.	Telehealth improves my access to health care services.
General benefits or value of virtual care	7 (50)	—	—
Acceptability	7 (50)	—	The way I interact with this system is pleasant. I like using the system.
Clear instructions	3 (21)	—	It was easy to learn to use the system. The system gave error messages that clearly told me how to fix problems.
Easy to use	7 (50)	I do not need assistance while using the system.	It was simple to use this system. I believe I could become productive quickly using this system, The system is simple and easy to understand. Whenever I made a mistake using the system, I could recover easily and quickly.
Provider competency with technology	2 (14)	—	—
Privacy and security	4 (29)	—	—
Overall satisfaction	11 (79)	Overall, I am satisfied with the quality of service being provided by telemedicine. I find telemedicine an acceptable way to receive health care services.	Overall, I am satisfied with this telehealth system. This system is able to do everything I would want it to be able to do. Telehealth is an acceptable way to receive health care services.
Comfort level during appointment	6 (43)	I feel comfortable communicating with my health care provider.	I feel comfortable communicating with the clinician using the telehealth system.
Audio or visual quality	6 (43)	I can hear my health care provider clearly. I can see my health care provider as if we met in person.	I can hear the clinician clearly using the telehealth system. Using the telehealth system, I can see the clinician as well as if we met in person.
Connectivity	7 (50)	—	—
Overall care quality	5 (36)	I do receive adequate attention.	—
Compare to in-person care	11 (79)	I think the health care provided by telemedicine is consistent.	I think the visits provided over the telehealth system are the same as in-person visits.
Care effectiveness	9 (64)	Telemedicine provides for my health care need.	Telehealth provides for my health care need.
Care provider quality	6 (43)	My health care provider is able to understand my health care condition.	—
Safety	2 (14)	—	—
Would you choose virtual care again?	10 (71)	I will use telemedicine services again.	I would use telehealth services again.
Recommend to others?	3 (21)	—	—
Dislikes, challenges	6 (43)	—	—
Areas for improvement	6 (43)	—	—

^a^Excluding demographics and visit details.

### Interviews

#### Overview

We conducted 6 follow-up interviews with respondents representing 5 institutions and several clinical areas: rehabilitation and development, ambulatory care, emergency medicine, mental health, and diabetes. The interview findings, including exemplar quotes, are presented in accordance with the a priori domains below. Where applicable, summarized interview findings are also presented alongside the questionnaire data in Tables 2 and 3 and Textbox 1.

#### Evaluation Approach

When selecting or developing their evaluation tools, many respondents conducted literature reviews and environmental scans; however, collaboration and consultation with peers or evaluation experts at their own and other institutions were also common. Among those that modified existing tools, the content was often changed to suit specific clinical contexts or patient populations, especially since many generic tools were designed for adult populations. As 1 respondent recalled:

We included some...open-ended [questions], and questions around what would have an in-person visit have cost you, how much time off work would you have missed for an in-person visit, and how much time off school would you have missed -- so capturing a little bit of what the impact of that visit is.

interview respondent 4

Some respondents consulted patient and family advisory councils and decision makers within their institutions when developing their evaluation tools to ensure they included relevant and meaningful questions. According to 1 respondent:

The biggest plug I would give is in terms of patient and family engagement. Having them really informed the work...having these evaluations be meaningful enough to decision makers within health systems. So [decision makers] would use these data to make decisions about whether they’re going to continue to fund virtual care...

interview respondent 5

#### Distribution Methods

The majority of respondents used email to distribute evaluation tools to children and youth, parents and caregivers, providers, and support staff. In some instances, health care providers and support staff were invited to complete evaluations during staff meetings or by team leaders or managers.

#### Response Rate

Response rates varied across evaluations. Some respondents reported being surprised by how high their response rate was, while others noted disappointment with low response rates, especially in 1 longitudinal evaluation where patients and families were intended to provide multiple responses. Many respondents felt that those who completed evaluations were representative of the population receiving or delivering virtual care, though some questioned whether their participant samples were representative of their broad or overall patient population.

Are the families who are responding, families who are more interested in the subject or more technologically adept?

interview respondent 4

So we [had] very, very positive folks and some folks [who] had really challenging experiences, but I think that’s the nature of people who tend to respond to requests to participate in surveys.

interview respondent 5

#### Lessons Learned

When reflecting on their evaluations, respondents noted several lessons learned. One respondent voiced that they would like to include a broader group of participants in the future to gain a wider variety of perspectives. The COVID-19 pandemic might provide more insight into how to plan for providing virtual care services in the future.

I [hope] to repeat the survey after COVID was less of a driver...we know that the pendulum swung way too far in the direction of doing virtual. It needs to swing a little bit more into the centre, so that patients are being seen using the right modality at the right time to meet their needs and preferences.

interview respondent 2

While web-based questionnaires seemed to work well for children, youth, and parents or caregivers, interviews and focus groups appeared to be a worthwhile choice for providers:

The best part and where we got the most information were the focus groups – I’m really happy that we did the focus groups.

interview respondent 3

One respondent also noted that it was easier to recruit and engage busy providers in-person instead of by email and speculated that in-person discussions were more appealing than web-based questionnaires for providers.

#### Core Set of Questions for Future Use

To conclude each interview, respondents were asked whether they felt a core set of commonly used questions for evaluating experience with virtual care would be useful to them in the future. Responses were unanimously positive in the interest of conserving time and resources needed to produce a new set of questions, though it was evident that a “validated tool” was preferred, as voiced by 2 respondents:

I would definitely be open to [it], especially if there were multiple groups using the same thing, being consistent and being able to then pull those responses...the preference is always to use a previously validated tool.

interview respondent 2

Of course a standardized, validated tool would be wonderful. But again, I think [it’s important to try] to limit the burden on the families in terms of the size and scope of all the questions that are being asked, and [make] sure that those data are being collected to serve the families.

interview respondent 4

Respondents also noted that although a core set of commonly used questions would be helpful, they may need to consider whether additional questions would be needed to suit their unique patient populations or clinical contexts.

## Discussion

### Principal Findings and Comparison to Previous Work

In this environmental scan, we conducted a web-based questionnaire and follow-up interviews to identify if and how evaluations of experiences with pediatric virtual care were being conducted in Canadian pediatric health care institutions. The most common method used by respondents to collect evaluation data was questionnaires distributed through email; 2 evaluations used validated tools, and the remainder modified existing tools or created new evaluation tools. The most common evaluation questions pertained to overall participant satisfaction, comparisons of virtual care to in-person care, and the likelihood of using virtual care again. Most evaluations targeted patients and families. Though some sought feedback from providers, very few were directed at support staff involved in virtual care delivery. Our interview findings describe the strengths and limitations of evaluation strategies and tools, and suggest that a standardized, core set of questions would facilitate data synthesis and an improved understanding of how virtual care should be delivered to children, youth, and families.

Despite the dramatic shift from in-person to virtual care during the COVID-19 pandemic, only 29 (40%) of the 72 respondents had started an evaluation. This suggests that while understanding virtual care experiences may be important to those involved in delivering pediatric virtual care, many have not had the opportunity to conduct an evaluation. This also suggests broad evaluation of virtual care experience in Canadian pediatric health care is lacking, which presents barriers to monitoring and improving the quality, accessibility, and acceptability of an overall care model that effectively integrates virtual care.

While all reported evaluations included parent or caregiver perspectives and most included patient and health care provider perspectives, few gathered the perspectives of support staff. Thus, few evaluations adhered to the Quintuple Aim [15], and this gap in understanding the experiences of nonclinical staff supporting virtual care programs arguably limits their ability to identify and address opportunities for improvement and optimize health system performance. The infrequency of questions related to education, race or ethnicity, and employment status further demonstrates that health equity, especially when evaluating a service that carries significant potential for accessibility barriers, has not been prioritized. Guidelines from the Canadian Institute of Health Information [26] and the Government of Canada’s Task Team on Equitable Access to Virtual Care [17] suggest that collecting sociodemographic information (eg, age, race or ethnicity, and place of residence) may be especially important when evaluating experiences with virtual care and could help identify barriers to positive experiences and contribute to improving supports for virtual care. Sociodemographic data can also help determine the representativeness of evaluation participants and understand the generalizability of findings. Future evaluations may benefit from this guidance and as suggested in our findings, incorporate unique patient, family, and other stakeholder perspectives to ensure methods and outputs are relevant, useful, and meaningful. Finally, satisfaction was one of the most frequently included question categories in the tools we assessed, and although satisfaction can reflect experience, it is only 1 part of the multidimensional and complex way that patients and families think about their health care [27]. In addition to satisfaction, exploring other concepts such as patient-centeredness, acceptability, and accessibility may help provide a more holistic view of patient experience.

Many respondents did not report or did not know the response rates for their evaluations. Among those who did, response rates ranged between 20% and 100%, but the high prevalence of missing data made patterns related to evaluation methods or target population difficult to discern. Understanding the effectiveness of respondents’ evaluation approaches was also limited for this reason.

When evaluating child, youth, and parent or caregiver experience with virtual care, all respondents in our study used questionnaires, and the majority distributed these through email. Though virtual care often requires patients and families to have access to email and technological literacy skills, evaluations conducted through email may not be equitable to individuals without these resources. Given that digital inclusion has been identified as a social determinant of health [28,29], varied evaluation approaches may be required to ensure responses are representative of the entire patient and family population.

Despite the frequent use of the TUQ, TSQ, and System Usability Scale in the literature [16,30], we found that only 1 evaluation in our sample used the unmodified TUQ, and this evaluation was not conducted in an exclusively pediatric setting. Some respondents suggested that previously developed tools were too generic and that questions specific to their clinical areas or patient populations were needed. Based on the literature and our findings, it appears that evaluators prefer to use readily available, validated tools, when possible, which underscores the importance of and need for a standard set of questions that could be used in pediatric virtual care evaluations going forward.

### Strengths and Limitations

This study has a number of strengths. By leveraging a wide network of health care providers and leaders and including the use of social media, we received responses from across Canada and a variety of clinical areas. All respondents who were approached for an interview agreed to participate. Each evaluation included in our analysis incorporated a unique tool and approach, which allowed for analysis of diverse methods to assess experiences with virtual care. As a result of incorporating qualitative interviews in our study design, we were able to gather detailed information about evaluators’ experiences that may not have been possible to collect by questionnaire alone.

There are some limitations to this environmental scan. First, despite a large number of completed questionnaires (N=72), we identified only 15 unique past or current evaluations. Thus, our final analysis was based on a relatively small number of evaluations. Second, we may not have captured all pediatric virtual care experience evaluations that occurred in Canada, either due to not reaching all groups who conducted evaluations, or to nonresponse, as our study was conducted during the COVID-19 pandemic and at a notably busy time of the year for health care providers and leaders. Third, most of our respondents represented large academic health care centers located in urban settings that are more likely to have the capacity to conduct evaluations. As such, we may have missed the perspectives of smaller, rural centers and Northern communities. However, the academic centers, including our own, which was well represented in this study, do include these populations in their service areas. In the future, we will incorporate a more targeted recruitment strategy in an effort to gain more proportionate responses from across Canada. We would also more clearly define our eligibility criteria to exclude those who had not conducted an evaluation or explore barriers to evaluating experiences among such respondents.

### Future Directions

We identified a clear appetite to improve virtual care evaluation strategies, especially as virtual modalities remain an option for many families accessing pediatric care. With the findings of this environmental scan in hand, we recommend some next steps for evaluating experiences with pediatric virtual care. Given that few evaluations were designed for, or had sufficient validity evidence to support, use in pediatric contexts, it is important to consider the unique aspects of pediatric care (eg, the involvement of parents or caregivers in children’s health care and privacy) when designing and conducting these evaluations. Furthermore, future evaluations should collect sociodemographic data when possible; the tools we analyzed lacked demographic questions, limiting the ability to assess whether the study sample is representative of the larger population. Drawing upon these results and available validated tools such as the TUQ and TSQ, we recommend the development of a core set of questions to evaluate experiences with pediatric virtual care. Such a tool will support standardized data collection, toward synthesis and meaningful comparisons of data across institutions and clinical contexts, to inform high-quality care models that are patient-centered, sustainable, and acceptable to all stakeholders. These findings and recommendations add to the growing body of literature [9,31-33] calling for a more standardized evaluation of virtual care.

## Appendix

Multimedia Appendix 1 CHERRIES (Checklist for Reporting Results of Internet E-Surveys).

Multimedia Appendix 2 Environmental scan questionnaire.

Multimedia Appendix 3 Information letter for survey.

Multimedia Appendix 4 Interview guide.

Multimedia Appendix 5 Questionnaire item summary.

## Abbreviations

CHERRIES: Checklist for Reporting Results of Internet E-Surveys
GRAMMS: Good Reporting of Mixed Methods Study
TSQ: Telehealth Satisfaction Questionnaire
TUQ: Telehealth Usability Questionnaire

## Data Availability

Data from this study are available upon reasonable request from the corresponding author.
