# Supplementary material for: Evaluation Strategies for Understanding Experiences With Virtual Care in Canada: Mixed Methods Study
Source: J Med Internet Res. 2023 Aug 30;25:e45287. doi: 10.2196/45287 (PMC10500354; doi:10.2196/45287)
Supplement: Multimedia Appendix 2 [file jmir_v25i1e45287_app2.docx]

**Multimedia Appendix 2: Environmental scan questionnaire.**

1. Which organization do you work for? ____________________
2. What best describes your role(s)? (select all that apply).

- Director
- Manager
- Program lead
- Practicing clinician
- Administrative role
- Researcher
- Other (if select other, please specify)

1. Please indicate the clinical area(s) you work in or oversee that provide care virtually either by telephone or video conference (select all that apply):

- Emergency Department / Urgent Care
- Critical Care (ICU, NICU)
- Inpatient Medicine (including subspecialties)
- Inpatient Surgery and Anesthesia
- Perioperative Services (outpatient surgery, day surgery, pre-assessment clinic)
- Outpatient Medical Clinics / Ambulatory Care (including Medical Day Unit)
- Inpatient Mental Health
- Outpatient Mental Health
- Inpatient Development and Rehabilitation
- Outpatient Development and Rehabilitation
- Genetics
- Autism
- Other (please specify_______________)

1. Of the areas you have indicated in the previous question, which ones have evaluated or are currently evaluating their virtual care (select all that apply)?

- Emergency Department / Urgent Care
- Critical Care (ICU, NICU)
- Inpatient Medicine (including subspecialties)
- Inpatient Surgery and Anesthesia
- Perioperative Services (outpatient surgery, day surgery, pre-assessment clinic)
- Outpatient Medical Clinics / Ambulatory Care (including Medical Day Unit)
- Inpatient Mental Health
- Outpatient Mental Health
- Inpatient Development and Rehabilitation
- Outpatient Development and Rehabilitation
- Genetics
- Autism
- Other (please specify_______________)
- Not sure

1. Please indicate for each group (patient, parent/caregiver, healthcare provider, support staff) whether you have evaluated or are evaluating their experience with virtual care (check all that apply):

|  | Evaluation complete | In progress | No evaluation started |
| --- | --- | --- | --- |
| Patient experience |  |  |  |
| Parent/caregiver experience |  |  |  |
| Healthcare provider experience |  |  |  |
| Support staff experience |  |  |  |

*If for patient experience they indicate “our evaluation is complete” then ask about dates and response rate as follows:

1. When did you start your patient experience evaluation? [month/year]
2. When did you complete your patient experience evaluation? [month/year]
3. What was the response or participation rate?

- 0-20%
- 21-40%
- 41-60%
- 61-80
- greater than 80%
- I don’t know

**Repeat for parent / caregiver experience, healthcare provider experience and support staff experience only if they select “Our evaluation is complete” for the corresponding group.

***If they pick “No evaluation started to date” for ALL four groups, then skip to Question 11.

[End of questionnaire for this participant]

1. Please indicate for each group (patient, parent/caregiver, healthcare provider, support staff) how virtual care experiences have been or are being evaluated (check all that apply):

**Note each row below only appears if the participant indicates an evaluation is complete or in progress for the group

|  | Survey interview | Focus group | Other |
| --- | --- | --- | --- |
| Patient experience |  |  |  |
| Parent/caregiver experience |  |  |  |
| Healthcare provider experience |  |  |  |
| Support staff experience |  |  |  |

*If they select “Other method” for Patient Experience then ask:

- Please specify the “other” method used to evaluate Patient Experience ________

**Repeat for all groups they select “Other method”

1. Please indicate for each group which type(s) of recruitment strategy you used or are using (check all that apply):

|  | Patient portal | Email | Phone call | Text/SMS | Social media | Other |
| --- | --- | --- | --- | --- | --- | --- |
| Patients |  |  |  |  |  |  |
| Parents/caregivers |  |  |  |  |  |  |
| Healthcare providers |  |  |  |  |  |  |
| Support staff |  |  |  |  |  |  |

*If Other selected, then ask:

“Please indicate which “Other” methods you are using to recruit participants for your evaluation(s)________________________________

1. How did you determine which questions to include in your evaluation(s)? Please select all that apply.

- Environmental scan (asking people in other departments, institutions, or organizations about their experiences)
- Literature review
- Consultation with experts/advisors
- Please explain your approach for consultation with experts / advisors _________
- Other

Please explain what other strategies you used to formulate your evaluation questions_______

1. Please indicate for each group what tool(s) you are using to evaluate virtual care experience(s) (check all that apply):

|  | Existing tool | Modified existing tool | Our own tool | N/A |
| --- | --- | --- | --- | --- |
| Patient experiences |  |  |  |  |
| Parent/caregiver experiences |  |  |  |  |
| Healthcare Provider experience  Support Staff experience |  |  |  |  |

1. Please provide any other information that would help us understand how you are evaluating virtual care at your institution (including key findings, or what you would change if you had to do it over again).

______________________________________________________________________________

1. May we contact you in future for any of the following:

- You may contact me to schedule a follow-up interview (30 minutes) to exchange knowledge on virtual care evaluation. [Yes/No]
- You may contact me to request a copy of evaluation tools, results or other documents we used to guide virtual care evaluation [Yes/No].
- You may contact me to share results from this study. [Yes/No]

*if they select Yes to one or more of the questions above ask the following

1. Name______________________
2. Email______________________
3. Confirm email_________________
