# Supplementary material for: Evaluation Strategies for Understanding Experiences With Virtual Care in Canada: Mixed Methods Study
Source: J Med Internet Res. 2023 Aug 30;25:e45287. doi: 10.2196/45287 (PMC10500354; doi:10.2196/45287)
Supplement: Multimedia Appendix 3 [file jmir_v25i1e45287_app3.docx]

## Multimedia Appendix 3: Information Letter for Survey

**Study title**: An Environmental scan of evaluation strategies to understand individual experiences with virtual care

**Principal Investigators**:

Catherine Dulude, Human Factors Specialist, Virtual Care Leadership Team Member, CHEO. [cdulude@cheo.on.ca](mailto:cdulude@cheo.on.ca)

Dr. Ellen Goldbloom, Pediatric Endocrinologist, Medical Lead Virtual Care, Interim Deputy CMIO, CHEO; Assistant Professor, Pediatrics, University of Ottawa. [egoldbloom@cheo.on.ca](mailto:egoldbloom@cheo.on.ca)

**You are invited to complete a survey for an environmental scan of how individual experiences with virtual care are being evaluated** by CHEO and health care organizations across Canada. This survey examines methodologies used for data collection and evaluation of individual experiences with virtual care. Operational, clinical, or research leads for any program that delivers care virtually are invited to participate. **The survey below will take about 5 to 15 minutes to complete.** We hope to engage 60 leaders from within CHEO and other health care organizations across Canada.

**This study is being done to** help determine the most frequently used and effective data collection strategies for evaluating individual experiences with virtual care. In particular, we are interested in the experiences of patients, caregivers, health care providers and support staff. This study is part of a larger Virtual Care Evaluation Project co-sponsored by CHEO and the CHEO Research Institute, which aims to develop an inclusive and cohesive virtual care evaluation framework, including the identification and development of tools to support virtual care program design and delivery.

**Taking part in this study is voluntary**. Your decision to participate or not will not affect your employment and is not related to performance evaluation in any way. You are free to withdraw from the study at any time and there will be no penalty. You may choose to stop participating in the study or withdraw any data previously submitted by sending an email to the Research Coordinator: Shelley Vanderhout at [svanderhout@cheo.on.ca](mailto:svanderhout@cheo.on.ca).

**By completing the survey below, you are agreeing to take part in this study.**

**If you prefer to participate through a virtual interview instead** of completing this survey, please contact Shelley Vanderhout at [svanderhout@cheo.on.ca](mailto:svanderhout@cheo.on.ca).

**If you feel another/other member(s) of your team would be better suited to complete the survey**, please forward the invitation email or survey link to them.

All information gathered from the surveys and any follow-up interviews will be kept strictly confidential. Information about respondents (role, contact information) will be collected so that we can describe the respondents as part of reporting. However, if we publish or present the study results, we will not use any identifying information. We will keep all survey data on the secure REDCap server managed by the CHEO Research Institute.

Data collected for this research may be used in future related research projects that are either an extension of the original project or in the same general area of research (secondary use of data). Any personal identifying information will be removed from the data and cannot be linked back to you. Researchers outside of this specific study may request access to the data for new research purposes. You will not be asked to provide additional informed consent for the use of your de-identified data for future research. Following completion of the research study the data will be kept for 10 years, after the last publication of this study. They will then be destroyed.

You may or may not directly benefit from the study. However, your input and perspective would be of tremendous value to us. Participation will make you eligible to receive a copy of the results, which you can use to help inform or improve your own virtual care evaluation strategy. The risks may include you feeling uncomfortable with some of the questions being asked. If you feel uncomfortable, you may choose not to answer a question. If you choose to participate in a virtual interview, please note the use of virtual platforms, like any internet communication or storage and retention of information, involve privacy risks around access and disclosure of information, however, there are safeguards in place to reduce these risks, (e.g., account registration, meeting passwords, disposal of records or devices on which information is stored).

Once the study is complete, we will share a summary report of the results with participants who indicate they wish to receive a copy of study results and provide their contact information.

There are no conflicts of interest to declare related to this study. The CHEO Research Ethics Board (REB) has reviewed and approved this study. Should you have any questions about your rights as a research participant, or ethical issues related to this study, you can talk to someone who is not involved in the study the CHEO REB at 613-737-7600 ext. 3272.

**Who to contact if you have questions about this study:**

Shelley Vanderhout at [svanderhout@cheo.on.ca](mailto:svanderhout@cheo.on.ca) or Catherine Dulude at [cdulude@cheo.on.ca](mailto:cdulude@cheo.on.ca).

Your assistance with this study is greatly appreciated. Thank you for your time and consideration.
