# Supplementary material for: Evaluation Strategies for Understanding Experiences With Virtual Care in Canada: Mixed Methods Study
Source: J Med Internet Res. 2023 Aug 30;25:e45287. doi: 10.2196/45287 (PMC10500354; doi:10.2196/45287)
Supplement: Multimedia Appendix 4 [file jmir_v25i1e45287_app4.docx]

**Multimedia Appendix 4: Interview guide.**

1. Based on your questionnaire response, I understand your target population was [patients/families/providers/support staff], and you used [surveys/interviews/focus groups/other] to find out about experiences with virtual care. Is that right? And the evaluation is [in progress/complete]?
2. [How did recruitment go/How is recruitment going?] Were you expecting that? Why or why not? What worked well about the recruitment strategy? What did not work so well/would you change if you were to do it again?
3. Did you feel that the people who participated in your evaluation were representative of the larger [patient/family/provider/support staff] population? Why or why not?
   1. Were there any biases in the responses?
   2. Are you aware of any equity- or access-related barriers to virtual care, which may have impacted who participated in your evaluation?
4. [if complete] Overall, would you consider your evaluation a success? Why or why not?
5. What would you do differently next time?
6. I also see that you used [tool/approach]. Why did your team choose that tool/approach? [if not sure, ask if validated, and whether they collected demographic questions]
7. Was there anything you learned through your evaluation that was particularly surprising or helpful for the improvement of your virtual care program or services?
8. If there were to be a core set of questions available to evaluate individual experiences with virtual care, would you be interested in using them in future evaluations?
9. Is there anything else you’d like to share about your experience conducting this evaluation?
