# Supplementary material for: Evaluation Strategies for Understanding Experiences With Virtual Care in Canada: Mixed Methods Study
Source: J Med Internet Res. 2023 Aug 30;25:e45287. doi: 10.2196/45287 (PMC10500354; doi:10.2196/45287)
Supplement: Multimedia Appendix 5 [file jmir_v25i1e45287_app5.docx]

## Multimedia Appendix 5: Questionnaire item summary.

| **Question domain** | **Question category** | **Count (N=14^a^)** | **Sample item** |
| --- | --- | --- | --- |
| **Demographics** | Intended audience/screening | 7 | Who is completing this survey?   Patient   Parent/Caregiver: mother, father, other |
|  | Age | 6 | What is the age of the child/youth who accessed services? |
|  | Sex or gender | 3 | Your child's gender:   Male  Female  Transgender Female   Transgender Male   Gender-variant/Non-Conforming   Not Listed  Prefer not to answer |
|  | Proximity to hospital | 4 | What is the distance from your home to the hospital? |
|  | Education level (caregiver/patient) | 1 | Your grade:   Grade 6   Grade 7   Grade 8   Grade 9   Grade 10   Grade 11   Grade 12   University/community college   I’m not in school   Other   Prefer not to answer |
|  | Ethnicity/race | 1 | What is your racial or ethnic background? |
|  | Virtual care exposure | 6 | *Caregivers/patients:* In the last 6 months, how many times have you used telehealth technologies with this healthcare provider?;  *Providers:* How long have you been providing virtual care services? |
|  | Family structure | 2 | Who else lives in your household? |
|  | Employment status | 1 | What is your employment status? |
|  | Access to technology | 3 | Type of technology available at home:   Smartphone   Tablet   Laptop/desktop   Internet reliability |
| **Visit details** | Reason for visit | 8 | What are the reasons you are using telehealth technologies with that provider? |
|  | Who attended visit | 3 | From your family, who attended the recent virtual visit?;  Who joined from your diabetes team? (select all that apply) □ Doctor □ Nurse  □ Dietitian □ Social worker □ Other health care provider(s)  □ Other |
|  | Mode | 6 | Visit type:   Telephone   Online video or Zoom   Both |
|  | Type of device | 2 | What type of device were you using for your virtual visit? |
|  | Preparation | 4 | I received instructions before my virtual visit that helped me prepare and know how to join the visit (strongly agree to strongly disagree) |
| **Benefits of virtual care** | Travel time | 8 | How many minutes of travel time (to and from) were you saved by attending a virtual visit as opposed to an in-person visit? |
|  | Money saved | 3 | Please estimate the dollar amount saved by having a virtual visit |
|  | Convenience | 4 | Virtual visits are convenient (strongly agree to strongly disagree) |
|  | Wait time (day of appointment) | 4 | Virtual care allows my child to receive care in a timely manner (strongly agree to strongly disagree) |
|  | Access to care | 5 | Telehealth improves my access to healthcare services (strongly agree to strongly disagree) |
|  | General benefits or value of virtual care | 7 | What are the benefits of having emergency virtual care services available to you, if any? |
| **Technology – Usability** | Acceptability | 7 | The way I interact with this system is pleasant (agree to disagree); Did you feel you were able to express yourself effectively? |
|  | Clear instructions | 3 | It was easy to learn to use the system (strongly agree to strongly disagree) |
|  | Easy to use | 7 | Was the virtual system simple and easy to understand? (not at all to completely) |
|  | Provider competency with technology | 2 | The staff knew how to work the technology (strongly agree to strongly disagree) |
|  | Privacy and security | 4 | I am confident that my health information was kept private and confidential (strongly agree to strongly disagree) |
|  | Overall satisfaction | 11 | What was your overall satisfaction with your telemedicine visit? (very unsatisfied to very satisfied) |
|  | Comfort level during appointment | 6 | Did you feel comfortable communicating with the doctor using the virtual system? (not at all to completely) |
| **Technology – Quality of connection** | Audio/visual quality | 6 | I am satisfied with the quality of virtual care audio and visual technology (strongly disagree to strongly agree) |
|  | Connectivity | 7 | Did you experience any difficulty connecting into the Zoom asthma education session? |
| **Care quality** | Overall care quality | 5 | *Caregiver/patient:* How would you describe the quality of the care delivery provided through participation in this program?  *Provider*: I feel confident in the quality of care I provide virtually (strongly agree to strongly disagree) |
|  | Compare to in-person care | 11 | My virtual visit experience was just as good or better than an in-person visit at the hospital (strongly agree to strongly disagree) |
|  | Care effectiveness | 9 | At the end of the visit, I understood the next steps in my care or treatment (strongly agree to strongly disagree);  Did you feel your child’s care is being compromised as a result of the telehealth/virtual care use? |
|  | Care provider quality | 6 | My physician provided all necessary information, including my child's diagnosis and plan, in an understandable way (strongly agree to strongly disagree) |
|  | Safety | 2 | I felt my child received safe and adequate care (strongly agree to strongly disagree) |
| **Future use** | Would you choose virtual care again? | 10 | Where possible, I would prefer that future in-person hospital appointments be completed as a virtual visit (completely agree to completely disagree) |
|  | Recommend to others? | 3 | I would recommend virtual health care to other people (strongly agree to strongly disagree) |
| **Challenges and improvements** | Dislikes, challenges | 6 | Can you describe any challenges that you have experienced using virtual care services? |
|  | Areas for improvement | 6 | In what ways virtual care could be improved? |
| **Miscellaneous** |  | 8 | Was your preference for receiving the care your child needs, either in-person, virtually, or by your own family doctor, taken into consideration when using [our services]?  An interpreter was needed for my virtual visit.  My [patient portal] was a useful tool for my recent virtual visit.  Did you require an urgent follow-up in-person visit or physical exam following your telemedicine visit? |

**^a^**One respondent was not able to provide a copy of the evaluation tool they used.
